# Sympathovagal Imbalance Contributes to Prehypertension Status and Cardiovascular Risks Attributed by Insulin Resistance, Inflammation, Dyslipidemia and Oxidative Stress in First Degree Relatives of Type 2 Diabetics

**DOI:** 10.1371/journal.pone.0078072

**Published:** 2013-11-12

**Authors:** Gopal Krushna Pal, Chandrasekaran Adithan, Palghat Hariharan Ananthanarayanan, Pravati Pal, Nivedita Nanda, Thiyagarajan Durgadevi, Venugopal Lalitha, Avupati Naga Syamsunder, Tarun Kumar Dutta

**Affiliations:** 1 Department of Physiology, Jawaharlal Institute of Post-graduate Medical Education and Research (JIPMER), Puducherry, India; 2 Department of Pharmacology, Jawaharlal Institute of Post-graduate Medical Education and Research (JIPMER), Puducherry, India; 3 Department of Biochemistry, Jawaharlal Institute of Post-graduate Medical Education and Research (JIPMER), Puducherry, India; 4 Department of Biochemistry, Pondicherry Institute of Medical Sciences, Puducherry, India; 5 Department of Medicine, Jawaharlal Institute of Post-graduate Medical Education and Research (JIPMER), Puducherry, India; CRCHUM-Montreal Diabetes Research Center, Canada

## Abstract

**Background:**

Though cardiovascular (CV) risks are reported in first-degree relatives (FDR) of type 2 diabetics, the pathophysiological mechanisms contributing to these risks are not known. We investigated the association of sympathovagal imbalance (SVI) with CV risks in these subjects.

**Subjects and Methods:**

Body mass index (BMI), basal heart rate (BHR), blood pressure (BP), rate-pressure product (RPP), spectral indices of heart rate variability (HRV), autonomic function tests, insulin resistance (HOMA-IR), lipid profile, inflammatory markers, oxidative stress (OS) marker, rennin, thyroid profile and serum electrolytes were measured and analyzed in subjects of study group (FDR of type 2 diabetics, n = 72) and control group (subjects with no family history of diabetes, n = 104).

**Results:**

BMI, BP, BHR, HOMA-IR, lipid profile, inflammatory and OS markers, renin, LF-HF (ratio of low-frequency to high-frequency power of HRV, a sensitive marker of SVI) were significantly increased (p<0.0001) in study group compared to the control group. SVI in study group was due to concomitant sympathetic activation and vagal inhibition. There was significant correlation and independent contribution of markers of insulin resistance, dyslipidemia, inflammation and OS to LF-HF ratio. Multiple-regression analysis demonstrated an independent contribution of LF-HF ratio to prehypertension status (standardized beta 0.415, p<0.001) and bivariate logistic-regression showed significant prediction (OR 2.40, CI 1.128–5.326, p = 0.002) of LF-HF ratio of HRV to increased RPP, the marker of CV risk, in study group.

**Conclusion:**

SVI in FDR of type 2 diabetics occurs due to sympathetic activation and vagal withdrawal. The SVI contributes to prehypertension status and CV risks caused by insulin resistance, dyslipidemia, inflammation and oxidative stress in FDR of type 2 diabetics.

## Introduction

Recently it has been reported that Asian Indian phenotype is exceptionally predisposed to develop diabetes because of strong familial aggregation and abrupt change in lifestyle [1]. As reported by World Health Organization (WHO), the total number of diabetic population was 171 million in 2000, which is projected to rise up to 366 million in 2030 [2]. India has been declared as the diabetic capital of world [3], [4]. It has been observed recently that the cardiovascular disease (CVD) and diabetes in developing countries are quite prevalent in the younger age group that has serious economic implications [4]. Diabetes shares several risk factors with CVD and the risk for CVD escalates with the co-occurrence of diabetes [4]. Therefore, early detection and treatment of diabetes and CVD, especially in younger population is among the key health policies worldwide [5]. The screening for diabetes and CVD in high risk population is a major strategy to achieve this goal [6]. The first degree relatives (FDR) of diabetic patients have been identified to have higher risk of developing diabetes compared to the general population [7]. Also, reports have confirmed increased cardiovascular (CV) risks and prevalence of CVD in this high risk population [8], [9].

Recently, decreased heart rate variability (HRV) and sympathovagal imbalance (SVI) have been reported to be associated with CV morbidities and mortalities [10], [11]. SVI has been attributed as the primary pathophysiologic basis of metabolic dysregulation in diabetes mellitus [12], [13]. An earlier report indicates modified autonomic balance with sympathetic hyperactivity in FDR of diabetic patients [14]. However, one report suggests that HRV is preserved in FDR of diabetics if they do not have metabolic complications [15]. The metabolic complications reported in FDR of diabetic patients are low-grade inflammation, insulin resistance and hyperlipidemia [16]–[18]. All these metabolic abnormalities are known risk factors for CVD [19]. Though autonomic imbalance has been reported in FDR of diabetics [14], [15] and inflammation, insulin resistance & dyslipidemia have been reported to induce SVI [20]–[23], to best of our knowledge no study has been conducted to date to assess the contribution of SVI to CV risks in FDR of type 2 diabetic patients. Though oxidative stress (OS) has recently been implicated in the causation of insulin resistance and diabetes [24], no study has assessed OS in FDR of type 2 diabetics. Therefore, in the present study, we have assessed the contribution of OS and other metabolic derangements to the SVI and CV risks in this high risk population.

Recently, spectral analysis of HRV has been established as a sensitive tool for assessment of autonomic functions in health and diseases [25]. Therefore, in the present study we have assessed the association of SVI with the CV risks in FDR of diabetic patients by analysing the spectral indices of HRV, in addition to the evaluation of autonomic function by conventional autonomic function tests (CAFT).

## Methods

This is a cross-sectional blinded study. Volunteers who participated in the study were not aware about which group they belonged to during the study, as the grouping was done after compiling the family history of diabetes. This study was conducted as part of hypertension research project. After obtaining the approval of Research Council and Institutional Ethics Committee, of Jawaharlal Institute of Post-graduate Medical Education and Research (JIPMER), Puducherry, India, 176 subjects were recruited from undergraduate and postgraduate courses of JIPMER of 2011–2012 batch. Written informed consent was obtained from all the participants prior to commencement of the study procedures. They were classified into two groups.

1. Control group (n = 104): Normal healthy subjects without family history of diabetes.
2. Study group (n = 72): Normal healthy first degree relatives (FDR) with family history of type 2 diabetes mellitus. Subject of study group (FDR with history of type 2 diabetics) was defined as the subject having either of the parents or siblings diagnosed to have type 2 diabetes mellitus for at least one year and receiving treatment for the same. This was done through questionnaires and interview.

Healthy subjects (subjects without illness) were included in the study. Subjects receiving any medication, subjects with history of diabetes, smoking, hypertension, and hypertensive patients receiving medication were excluded from the study.

### Recording of Anthropometric and Basal CV Parameters

Subjects were asked to report to autonomic function testing (AFT) laboratory of Physiology Department at about 9 AM following a light breakfast, without tea or coffee. After obtaining the written informed consent, their age, height and body weight were recorded and body mass index (BMI) was calculated. The temperature of AFT laboratory was maintained at 25°C for all the recordings. As per the procedure of blood pressure (BP) recording recommended by joint national committee (JNC-7) on classification of BP [26], after twenty minutes rest in upright sitting position on an armed chair, systolic blood pressure (SBP), diastolic blood pressure (DBP) and basal heart rate (BHR) were recorded using Omron (SEM 1 Model) automatic BP monitor (Omron Healthcare Co. Ltd, Kyoto, Japan), and mean arterial pressure (MAP) was calculated. Rate pressure product (RPP) was calculated using the formula, RPP = systolic pressure×heart rate×10^−2^ [27].

The category of ‘prehypertension’ or ‘normotension’ was decided based on the level of BP of the subjects. When SBP was >120 mm Hg or DBP was >80 mmHg or both, the subject was considered as prehypertensive, as per the JNC-7 classification of BP [26]. Prehypertension status was calculated using the Statistical Package for the Social Sciences (SPSS). For the purpose, depending on the BP level, the 0 and 1 values were assigned to normotensives and prehypertensives respectively for consideration of the prehypertension status. Accordingly, the groups 0 and 1 were categorized for determination and analysis of prehypertension status in both control and study groups by the SPSS software.

### Recording of HRV

After 15 minutes of supine rest on a couch in AFT lab, ECG was recorded for 10 minutes for short-term HRV analysis following the standard procedure as recommended by Task Force on HRV [28]. For the purpose, ECG electrodes were connected and Lead II ECG was acquired at a rate of 1000 samples/second during supine rest using BIOPAC MP 100 data acquisition system (BIOPAC Inc., Goleta, CA, USA). The data was transferred from BIOPAC to a windows-based PC with AcqKnowledge software version 3.8.2. Ectopics and artefacts were removed from the recorded ECG. HRV analysis was done using the HRV analysis software version 1.1 (Bio-signal Analysis group, Kuopio, Finland). Frequency domain indices such as total power (TP) of HRV, normalized low-frequency power (LFnu), normalized high-frequency power (HFnu), ratio of low-frequency to high-frequency power (LF-HF ratio) and time-domain indices such as mean heart rate (Mean RR), square root of the mean squared differences of successive normal to normal intervals (RMSSD), Standard deviation of normal to normal interval (SDNN), the number of interval differences of successive NN intervals greater than 50 ms (NN50) and the proportion derived by dividing NN50 by the total number of NN intervals (pNN50) were calculated.

### Conventional Autonomic Functions Tests (CAFTs)

Three CAFTs were performed following the standard procedures [29].

#### Lying to standing test (HR and BP response to standing)

The BP and ECG were recorded in supine position. The subject was instructed to attain standing posture in 3 seconds. The ECG was continuously recorded during the procedure. The BP was recorded every 40 seconds by automatic BP monitor (Omron, SEM-1, Kyoto, Japan) till 5^th^ min. The 30∶15 ratio (ratio of maximum RR interval at 30^th^ beat to minimum RR interval at 15^th^ beat following standing) was calculated.

#### Deep breathing test (HR response to deep breathing)

The subject in sitting posture, HR and respiration monitoring was done from ECG recording and stethographic respiratory tracings recorded on the multichannel polygraph (Nihon-Kohden, Kyoto, Japan). A baseline recording of ECG and respiration was taken for 30 seconds. The subject was asked to take slow and deep inspiration followed by slow and deep expiration such that each breathing cycle lasted for 10 seconds, consisting of six breathing cycles per minute. The E∶I ratio (ratio of average RR interval during expiration to average RR interval during inspiration in six cycles of deep breathing) was calculated from ECG tracing.

#### Isometric handgrip test (BP response to isometric handgrip)

The baseline BP was recorded. The subject was asked to press handgrip dynamometer at 30% of maximum voluntary contraction for 2 minutes. The BP was recorded at 1^st^ minute and 2^nd^ minute of contraction. ΔDBP_IHG_ (maximum rise in diastolic BP above baseline) was noted.

### Measurement of Biochemical Parameters

Five ml of fasting blood sample was collected. The serum was separated from the blood samples of all the subjects for estimation of biochemical parameters. Free tri-iodothyronine (fT3), free thyroxine (fT4), thyroid stimulating hormone (TSH) and insulin were assayed by chemiluminiscence method using the kits of Siemens Healthcare Diagnostics Inc. (Tarrytown, NY, USA). Fasting blood glucose (FBG) was estimated by glucose oxidase method using glucometer (LifeScan Inc, Milpitas, CA, USA). For determination of insulin resistance, homeostatic model assessment of insulin resistance (HOMA-IR) was calculated by using the formula, HOMA-IR = FBS(mMol)×Insulin(μIU/L)/22.5.

Lipid profile (total cholesterol, triglycerides, high-, low- and very low-density lipoproteins) and serum electrolytes (Na^+^, Cl^−^, K^+^ and Ca^++^) were assessed using fully automated chemistry analyzer (AU400, Olympus, Orlando, FL, USA). Atherogenic index (AI) was calculated using the formula, log_10_(TG/HDL). The high-sensitive C-reactive protein (hsCRP) was estimated by enzyme immunoassay method using ELISA kit (dbc Diagnostics Biochem Canada Inc, Ontario, Canada). Interleukin-6 (IL6) was measured by enzyme immunoassay method using ELISA kit (Ani Biotech Oy, Tiilitie, Finland). Tumor necrosis factor-α (TNFα) was estimated by enzyme immunoassay method using ELISA kit (Ani Biotech Oy, Tiilitie, Finland). Oxidative stress was assessed by estimating thiobarbituric acid reactive substance (TBARS) using ELISA kit (Cayman Chemical Co., Ann Arbor, MI, USA). Renin was estimated by enzyme immunoassay method using the ELISA kit of DRG Diagnostics (DRG Instruments GmbH, Frauenbergstr, Marburg, Germany).

### Statistical Analysis of Data

SPSS version 13 (SPSS Software Inc., Chicago, IL, USA) and GraphPad InStat softwares (GraphPad Software Inc., San Diego, CA, USA) were used for statistical analysis. All the data were presented as mean±SD. Normality of data was tested by Kolmogorov Smironov test. For parametric data, the level of significance between the groups was tested by Student's unpaired ‘*t*’ test and for nonparametric data, the Welch's corrected *t* test was used. The association between LF-HF ratio with BMI, BHR, blood pressure parameters, HOMA-IR, lipid parameters, inflammatory markers and TBARS was assessed by Pearson's correlation analysis. The independent contribution of various parameters to LF-HF ratio was assessed by multiple regression analysis. The parameters that had significant association with LF-HF ratio in Pearson's correlation were considered for multiple regression. Multiple regression analysis was applied to evaluate the effects of independent variables such as BMI, markers of insulin resistance, inflammation, oxidative stress and prehypertension status on LF-HF ratio, the dependant variable. The prediction of LF-HF of HRV to increased RPP was assessed by bivariate logistic regression. As higher level of RPP has been documented as an established CV risk, and LF-HF ratio of HRV as the primary marker of SVI, LF-HF ratio was considered as the independent variable to assess impact of SVI on CV risk due to increased RPP, the dependent variable in the logistic regression analysis. The p value less than 0.05 was considered statistically significant.

## Results

### Age, BMI and CV Parameters

There was no significant difference in age (p = 0.4419) between the subjects of control group and study group (Table 1). The BMI, BHR, SBP, DBP, MAP and RPP of study group subjects were significantly more (p<0.0001) compared to that of control group subjects (Table 1).

**Table 1:** Comparison of age, body mass index (BMI), cardiovascular (CV) parameters, frequency domain indices (FDI) and time domain indices (TDI) of heart rate variability (HRV) and conventional autonomic function testing (CAFT) parameters of control group (subjects with no family history of diabetes) and study group (first degree relatives of type 2 diabetics) subjects.

Parameters	Control Group (n = 104)	Study group (n = 72)	P-Value
Age (years)	20.95±2.56	21.28±3.10	0.4419
BMI (kg/m^2^)	23.30±3.64	26.95±3.50	<0.0001
**Cardiovascular Parameters**			
BHR (per min)	70.80±8.68	81.35±9.76	<0.0001
SBP (mmHg)	110.20±6.86	119.84±7.32	<0.0001
DBP (mmHg)	69.70±5.78	78.30±6.60	<0.0001
MAP (mmHg)	83.30±6.55	92.24±6.40	<0.0001
RPP(mmHg/min)	78.10±7.36	97.50±8.20	<0.0001
**FDI of HRV**			
TP (ms^2^)	940.60±245.26	656.40±194.75	<0.0001
LFnu	40.12±15.52	58.40±18.16	<0.0001
HFnu	59.24±17.34	42.12±14.60	<0.0001
LF∶HF ratio	0.67±0.31	1.41±0.68	<0.0001
**TDI of HRV:**			
Mean RR (s)	0.847±0.12	0.737±0.11	<0.0001
RMSSD (ms)	60.30±20.70	38.35±14.60	<0.0001
SDNN (ms)	45.26±18.25	30.42±11.42	<0.0001
NN50	50.78±18.65	28.40±10.20	<0.0001
pNN50	30.67±13.10	20.60±8.90	<0.0001
**CAFT parameters**			
E∶I ratio	1.45±0.28	1.25±0.25	<0.0001
30∶15 ratio	1.27±0.23	1.40±0.20	<0.0001
ΔDBP_IHG_	20.18±5.35	26.82±6.20	<0.0001

Data expressed as mean±SD. P value<0.05 was considered significant. BMI: Body mass index; BHR: Basal heart rate; SBP: Systolic blood pressure; DBP: Diastolic blood pressure; MAP: Mean arterial pressure; RPP: Rate-pressure product; TP: Total power; LFnu: Normalized low-frequency power; HFnu: Normalized high-frequency power; LF-HF ratio: Ratio of low-frequency to high- frequency power; Mean RR: Mean heart rate; RMSSD: The square root of the mean of the sum of the squares of the differences between adjacent NN intervals; SDNN: Standard deviation of normal to normal interval; NN50: The number of interval differences of successive NN intervals greater than 50; pNN50: The proportion derived by dividing NN50 by the total number of NN intervals; E∶I ratio: The ratio of average RR interval during expiration to that of during inspiration in six cycles of deep breathing; 30∶15 ratio: The ratio of maximum RR interval at 30^th^ beat to minimum RR interval at 15^th^ beat following standing; ΔDBP_IHG_: The maximum rise in DBP above baseline following 30% of maximum voluntary contraction by isometric handgrip method.

### HRV and CAFT Parameters

Among the frequency domain indices of HRV, TP, and HFnu were significantly reduced (p<0.0001) and LFnu and LF-HF ratio were significantly increased (p<0.0001) in study group subjects compared to the control group subjects. All the time domain indices (mean RR, RMSSD, SDNN, NN50, pNN50) were significantly less (p<0.0001) in study group subjects compared to that of control group subjects. The E∶I ratio was significantly decreased, and ΔDBP_IHG_ and 30∶15 ratio were significantly increased (p<0.0001) in study group subjects (Table 1).

### Metabolic Biomarkers

FBG, insulin and HOMA-IR were significantly increased (p<0.0001), all lipid parameters (except HDL, which was decreased) and lipid risk factors were significantly increased (p<0.0001) in study group compared to the control group (Table 2). Inflammatory markers (IL6, hsCRP, TNFα), renin and TBARS were significantly increased (p<0.0001) in study group compared to the control group. There was no significant difference in thyroid profile parameters between the groups. Serum Na^+^, Cl^−^, Ca^++^ levels were increased and K^+^ level was decreased (p<0.0001) in study group compared to the control group (Table 2).

**Table 2:** Comparison of metabolic biomarkers between of control group (subjects with no family history of diabetes) and study group (first degree relatives of type 2 diabetics) subjects.

Parameters	Control Group (n = 104)	Study group (n = 72)	P-Value
**Insulin related parameters:**			
FBG (mg/dL)	72.35±8.60	98.42±10.50	<0.0001
Insulin (μIU/mL)	5.24±2.40	18.74±5.58	<0.0001
HOMA-IR	1.30±0.75	4.56±1.60	<0.0001
**Lipid related parameters**			
TC (mg/dL)	150.10±25.60	186.70±27.54	<0.0001
TG (mg/dL)	90.28±22.41	142.68±30.20	<0.0001
LDL (mg/dL)	96.50±20.80	130.37±26.60	<0.0001
VLDL (mg/dL)	18.15±5.12	28.60±7.20	<0.0001
HDL (mg/dL)	45.20±8.70	35.46±6.90	<0.0001
TC/HDL	3.30±1.20	5.28±1.50	<0.0001
TG/HDL	2.02±0.86	4.10±1.20	<0.0001
LDL/HDL	2.15±0.92	3.70±1.08	<0.0001
Atherogenic index (AI)	0.35±0.13	0.59±0.15	<0.0001
**Inflammatory markers**			
IL6 (pg/mL)	30.22±10.56	92.45±25.20	<0.0001
hsCRP (ng/dL)	540.40±120.30	986.80±150.20	<0.0001
TNFα (pg/mL)	75.20±24.76	294.36±50.80	<0.0001
**Oxidative stress marker:**			
TBARS (µM/L)	2.05±0.60	4.14±1.10	<0.0001
**Sympathetic marker:**			
Renin (pg/mL)	35.36±13.46	72.80±15.50	<0.0001
**Thyroid profile**			
fT3 (pg/mL)	3.50±1.96	4.05±2.10	0.077
fT4 (ng/dL)	2.58±1.55	3.10±1.96	0.051
TSH (μIU/mL)	2.86±2.10	2.44±1.85	0.163
**Serum Electrolytes**			
Na^+^ (mEq/L)	137.10±6.28	145.30±7.50	<0.0001
K^+^ (mEq/L)	4.15±0.58	3.50±0.42	<0.0001
Cl^−^ (mEq/L)	97.30±7.54	108.87±10.70	<0.0001
Ca^++^ (mEq/L)	9.24±0.74	9.92±1.10	<0.0001

Data expressed as mean±SD. AI = Log10(TG/HDL); HOMA-IR = FBG(mMol)×Insulin(μIU/L)/22.5; FBG: Fasting blood glucose; HOMA-IR: homeostatic model assessment of insulin resistance; TC: Total serum cholesterol; TG: Triglyceride; LDL: Low-density lipoprotein; VLDL: Very low-density lipoprotein; HDL: High-density lipoprotein; IL6: Interleukin 6; hsCRP: high-sensitive C reactive protein; TNFα: Tumor necrosis factor α; TBARS: Thiobarbituric acid reactive substance; fT3: free tri-iodothyronine, fT4: free thyroxine; TSH: thyroid stimulating hormone.

### Correlation and Regression Analysis

Though there was no significant correlation of LF-HF ratio with any of the parameter in control group, the correlation of BMI, CV parameters and metabolic factors with LF-HF ratio was significant in the study group (Table 3).

**Table 3.Correlation:** Correlation of LF-HF with various parameters in both the groups.

Parameters	Control Group (n = 104)		Study group (n = 72)	
	r	p	r	p
BMI	0.086	0.182	0.254	0.038
BHR	0.097	0.155	0.365	0.004
SBP	0.110	0.120	0.325	0.012
DBP	0.102	0.132	0.270	0.024
MAP	0.128	0.092	0.340	0.008
RPP	0.072	0.186	0.342	0.008
HOMA-IR	0.156	0.068	0.710	<0.001
TC/HDL	0.092	0.106	0.530	<0.001
TG/HDL	0.072	0.186	0.538	<0.001
LDL/HDL	0.065	0.210	0.435	0.001
Atherogenic index	0.150	0.060	0.576	<0.001
IL6	0.076	0.180	0.659	<0.001
hsCRP	0.041	0.286	0.662	<0.001
TNFα	0.130	0.080	0.682	<0.001
Renin	0.115	0.109	0.350	0.007
TBARS	0.032	0.310	0.272	0.025

The p value <0.05 was considered significant. BMI: Body mass index; BHR: Basal heart rate; SBP: Systolic blood pressure; DBP: Diastolic blood pressure; MAP: Mean arterial pressure; RPP: Rate-pressure product; HOMA-IR: homeostatic model assessment of insulin resistance; TC: Total serum cholesterol; TG: Triglyceride; LDL: Low-density lipoprotein; HDL: High-density lipoprotein; IL6: Interleukin 6; hsCRP: high-sensitive C reactive protein; TNFα: Tumor necrosis factor α; TBARS: Thiobarbituric acid reactive substance.

Multiple regression analysis revealed significant individual contribution of HOMA-IR, AI, IL6, hsCRP, TNFα, TBARS and hypertension status, but not BMI to the LF-HF ratio in the study group (Table 4). Bivariate logisitic regression (Table 5) showed significant prediction of LF-HF ratio of HRV to RPP in study group (OR 2.40, CI 1.128–5.326; p = 0.002) compared to that of control group (OR 0.62, CI 0.446–2.352, p = 1.085).

**Table 4:** Multiple regression analysis of LF-HF (as dependent variable) with various parameters (as independent variables) in study group subjects.

Independent Variables	Standardized Regression	95% C.I.		p values
		Lower bound	Upper bound	
BMI	0.102	−0.394	0.510	0.086
HOMA –IR	0.256	0.027	0.178	0.002
AI	0.132	0.001	0.042	0.038
IL6	0.156	0.002	0.032	0.016
hsCRP	0.134	0.000	0.001	0.030
TNFα	0.356	0.002	0.005	<0.001
TBARS	0.168	−0.044	0.158	0.011
PHTN status	0.415	0.003	0.008	<0.001

P values<0.05 considered significant. BMI: Body mass index; HOMA-IR: homeostatic model assessment of insulin resistance; AI: Atherogenic index; IL6: Interleukin 6; hsCRP: high-sensitive C reactive protein; TNFα: Tumor necrosis factorα; TBARS: Thiobarbituric acid reactive substance; PHTN status: Prehypertension status.

**Table 5:** Bivariate logistic regression analysis of RPP (as dependent variable) with LF-HF ratio of heart rate variability (as independent variable) in control group and study group subjects after adjusting for BMI.

	Control group		Study group	
	OR (95% C.I.)	p value	OR (95% C.I.)	p value
LF-HF ratio	0.62 (0.446 to 2.352)	1.085	2.40 (1.128 to 5.326)	0.002

p<0.05 considered significant; OR : odds ratio; RPP: rate pressure product; LF-HF: Ratio of low-frequency to high-frequency power of heart rate variability; C.I. : confidence interval.

## Discussion

### HRV and CAFT Parameters

In the study group, LF-HF ratio was significantly increased (p = 0.000) compared to the control group (Table 1) indicating a considerable enhancement in sympathetic activity in FDR of type 2 diabetics as increase in LF-HF ratio represents accentuation of sympathetic activity and decrease in this ratio represents facilitation of parasympathetic activity [25], [28]. As there was decrease in HF_nu_ in FDR of type 2 diabetics, SVI in these subjects is due to decreased parasympathetic activity as decrease in HF_nu_ represents decreased vagal modulation of cardiac drive [25], [28]. SVI in study group subjects could also be due to increase in sympathetic activity as there was increased LF_nu_, which reflects both sympathetic and parasympathetic drives to the heart [25], [28]. These findings corroborate with the reports of earlier studies [14], [15]. In addition, increase in ΔDBP_IHG_ in study group subjects indicates increased sympathetic reactivity in FDR of type 2 diabetics [29].

The TP of HRV was significantly decreased in study group subjects (Table 1) representing a substantial decrease in HRV in FDR of type 2 diabetics as TP represents the quantum of HRV spectrum [25], [28]. The TP not only represents the magnitude of HRV, but also the overall vagal drive of cardiac modulation [25], [28]. Therefore, decreased TP of HRV has recently been observed to be associated with sudden cardiac death and cardiac morbidities [10], [11], [30], [31]. Thus, decreased TP in FDR of diabetics could predispose them to adverse CV events. This was further supported by significantly high BHR in these subjects (Table 1). Resting heart rate is an index of vagal tone [29], and increased BHR has recently been reported to be associated with increased CV risks [32]. The time-domain indices of HRV were significantly reduced in study group (Table 1) that further confirms decreased vagal tone in FDR of diabetics, as time-domain indices represent parasympathetic modulation of cardiac activity [25], [28]. Further, increased 30∶15 ratio and decreased E∶I ratio in these subjects (Table 1) reflect decreased vagal reactivity in FDR of type 2 diabetics [29].

### Contribution of Metabolic Biomarkers to SVI

Though the exact cause of SVI can not be ascertained from the present study, insulin resistance could be a plausible mechanism as fasting blood glucose, plasma insulin and HOMA-IR were significantly high in study group (Table 2). Further, HOMA-IR was significantly correlated with LF-HF ratio in study group subjects (Table 2) and had significant independent contribution to LF-HF in these subjects (Table 4). As insulin resistance has been reported to cause autonomic imbalance (23), insulin resistance could contribute to SVI in FDR of type 2 diabetics. Further, these subjects are more prone to develop diabetes mellitus.

Low-grade inflammation has been reported in FDR of type 2 diabetics [16], and inflammatory markers such as hsCRP, IL6 and TNFα have been reported to induce SVI in various stress related disorders [20]. In the present study, not only IL6, hsCRP and TNFα were significantly high in study group (Table 2), but also they were significantly correlated with LF-HF ratio (Table 3). Moreover, each inflammatory marker had independent contribution to LF-HF ratio (Table 4), and TNFα had the highest influence. Thus, it appears that retrograde inflammation has strong contribution to SVI in FDR of type 2 diabetics, in which TNFα plays a pivotal role.

There are reports of dyslipidemia in FDR of type 2 diabetics [17], [18]. In the present study, all lipid profile parameters (except HDL) and lipid risk factors were significantly high in study group (Table 2) and lipid risk factors were significantly correlated with LF-HF ratio (Table 3). Moreover, in multiple regression model, AI had significant contribution to LF-HF ratio in study group subjects (Table 4), indicating that atherogenic lipid risk factors contribute to SVI in them. From among the lipid risk factors assessed in the present study, we selected AI for the regression model, as AI has recently been reported to be the better indicator of CV risk [33]. Increased plasma level of TG is reported to produce OS [34], and OS is known to induce SVI [35]. In the present study, TBARS was significantly more in study group (Table 2) and had significant correlation with LF-HF (Table 3). Moreover, TBARS had independent contribution to LF-HF in these subjects (Table 4). Thus, OS could be the link between the dyslipidemia and SVI in FDR of type 2 diabetics.

### Influence of BMI

Obesity has been reported to be more prevalent in individuals with family history of diabetes [15]. In the present study, BMI was significantly high in study group (Table 1), and had significant correlation with LF-HF ratio (Table 3). However, in multiple regression analysis, BMI had no independent contribution to LF-HF ratio. Thus, increased BMI in FDR of type 2 diabetics is unlikely to be a potential contributor to SVI in these subjects. Salamin et al have recently reported that although BMI and subcutaneous adiposity are not associated with cardiac parasympathetic indices of HRV, visceral adiposity contributes to decreased HRV [36]. Therefore, future research warrants estimation of visceral fat and its correlation with SVI to assess the contribution of visceral adiposity to CV risks in FDR of type 2 diabetics.

### RPP as CV risk

RPP is a measure of myocardial work load and oxygen consumption and increased RPP has been documented a CV risk [27]. In the present study, RPP was not only significantly increased in study group compared to the control group (Table 1), but also was significantly correlated with LF-HF ratio of HRV (Table 3). Therefore, it is expected that SVI in FDR of type 2 diabetics is linked to the myocardial stress, which could be an impending CV risk. As obesity per se is a CV risk factor, we assessed the independent association of RPP with LF-HF ratio by logistic regression adjusted for BMI (Table 5) and we found that LF-HF ratio significantly predicts RPP (OR, 2.40; CI, 1.128 to 5.326; p = 0.002) in these subjects. Thus, it appears that SVI in FDR of type 2 diabetics contributes to CV risks independent of BMI. Further, LF-HF ratio had independent contribution to prehypertension status as shown by multiple regression (Table 4), further indicating the increased CV risk in these subjects.

### Link of SVI to CV risks

Prediabetes, prehypertension and insulin resistance in adolescents and young adults remain for a longer duration exposing them to premature CV risks before clinically manifesting as full blown diabetes and hypertension during their adulthood [37]. In the present study, in young FDR of type 2 diabetics, insulin resistance, atherogenic lipid profile, low-grade inflammation and oxidative stress were found to contribute to SVI. Findings of the present study indicate that SVI is the major link between the metabolic derangements and CV risks and chronic SVI could be the physiological basis for future CV morbidities in FDR of type 2 diabetics. However, further research warrants the evaluation of the impact of SVI achieved by various non-pharmacological means on reduction of prehypertension status and CV risks in these high risk young individuals, as practice of yoga and slow breathing exercises have been reported to decrease sympathetic discharge, increase vagal tone and improve metabolic functions [38], [39].

In summary, the results of the present study indicate the presence of SVI in the form increased sympathetic and decreased parasympathetic activity and reactivity in young FDR of type 2 diabetics. Resting tachycardia, decreased HRV, increased RPP, increased prehypertension status, insulin resistance, atherogenic lipid profile, low-grade inflammation and oxidative stress in these subjects make them vulnerable to augmented CV risks and SVI is the physiological basis for development of these CV risks. The present study suggests for future research to assess if restoration of sympathovagal homeostasis in FDR of type 2 diabetics can reduce the CV risks in these subjects.

### Limitations of the Study

In the present study, though we have assessed the CV risks, we have not assessed CV functions by continuous BP variability analysis to determine CV reactivity and dysfunction. We have also not estimated plasma norepinephrine or its metabolites in urine to support SVI. We have not performed analysis of body fat composition to assess the influence of visceral fat on SVI in FDR of type 2 diabetics.
